# Toxicological evaluation of microbial secondary metabolites in the context of European active substance approval for plant protection products

**DOI:** 10.1186/s12940-024-01092-0

**Published:** 2024-06-04

**Authors:** Norman Paege, Sabrina Feustel, Philip Marx-Stoelting

**Affiliations:** German Federal Institute for Risk Assessment (BfR), Berlin, Germany

**Keywords:** Plant protection active substance, Beauvericin, 2,3-deepoxy-2,3-didehydro-rhizoxin, Leucinostatin A, Swainsonin, Microbial secondary metabolites

## Abstract

Risk assessment (RA) of microbial secondary metabolites (SM) is part of the EU approval process for microbial active substances (AS) used in plant protection products (PPP). As the number of potentially produced microbial SM may be high for a certain microbial strain and existing information on the metabolites often are low, data gaps are frequently identified during the RA. Often, RA cannot conclusively clarify the toxicological relevance of the individual substances. This work presents data and RA conclusions on four metabolites, Beauvericin, 2,3-deepoxy-2,3-didehydro-rhizoxin (DDR), Leucinostatin A and Swainsonin in detail as examples for the challenging process of RA. To overcome the problem of incomplete assessment reports, RA of microbial AS for PPP is in need of new approaches. In view of the Next Generation Risk Assessment (NGRA), the combination of literature data, *omic*-methods, in vitro and in silico methods combined in adverse outcome pathways (AOPs) can be used for an efficient and targeted identification and assessment of metabolites of concern (MoC).

## Introduction

To reduce the potentially negative effects of chemical substances on human health and the environment, the EU presented the "European Green Deal" and the “Chemical Strategy for Sustainability” [40]. Critical aspects of this strategy from a scientific perspective were summarized by [60]. The Green Deal also comprises the “Farm to Fork Strategy” which summarizes developments "*Towards a fair, healthy and environmentally friendly food system*" [40, 41]. One goal is to reduce the use of chemical pesticides by 50% by 2030 [41]. As one way to fill the resulting lack of active substances is the approval of biopesticides, an increase in the submission of applications for microbial strains is expected, either for new approvals or the renewal of existing approvals. As one way to support and facilitate the approval of microorganisms revised data requirements for microbial active substances as well as plant protection products have been published recently by the EU Commission [21, 22]. Similarly, the competent authorities are endeavouring to streamline and accelerate the approval of AS and the authorization of PPPs by the revision of uniform EU-wide templates and procedural descriptions. A major contribution for the EU-wide uniform assessment of microbial secondary metabolites was the preparation of a guidance document for the identification and the assessment of toxicologically relevant secondary metabolite produced by microbial AS. In case such a toxicologically relevant metabolite is identified the revised data requirements clearly indicated that it has to be further assessed according to Part A of Regulation 283/2013, comparable to a conventional chemical AS or its metabolite [93].

Microbial biopesticides include viruses, bacteria or fungi (or only components thereof) as active ingredients that will prevent or reduce the adverse effects a pest can have on plants or plant products [6]. The approval of microbial AS and the authorization of PPP in the supranational association of states of the European Union is governed by Regulation (EU) 1107/2009 in compliance with Regulation (EU) 2022/1439 (amending Regulation (EU) No 283/2013 as regards the information to be submitted for microbial AS), Regulation (EU) 2022/1440 (amending Regulation (EU) No 284/2013 as regards the information to be submitted for plant protection products containing microorganisms) and Regulation (EU) 2022/1441, (amending Regulation (EU) No 546/2011 as regards specific uniform principles for evaluation and authorisation of plant protection products containing microorganisms).

In brief, an applicant that wants to market a microbial AS has to submit a dossier containing data on the safety of the microbial AS to an EU member state competent authority. The member state acts as the rapporteur member state (RMS) and prepares a dossier on the basis of the data provided by the applicant which serves – after commenting through other MS—as the basis for the Conclusion report of the European Food Safety Authority (EFSA). In this report the results of the risk assessment process as well as the identified data gaps are summarized. Based on EFSA's conclusions, the assessment of the RMS and other legitimate factors, the Commission submits a draft regulation for approval or non-approval and an accompanying review report. The Standing Committee on Plants, Animals, Food and Feed (ScoPAFF) votes on the decision of the approval (or non-approval if risks are not acceptable) of the AS and the associated risk management measures, which are then endorsed by the European Commission (EC) [39]. In contrast to the AS approval, PPP are approved at national level either by a single member state, or for a group of MS in one zone. A granted authorization can be accepted in other countries of the EU based on a process called mutual recognition. More details on the approval and authorization procedure are published on the EU website (https://ec.europa.eu). In principle, microbial biopesticides must not be pathogenic or infectious in order to be approved and miss transferable antibiotic resistance genes (Regulation (EU) No. 546/2011, C 2.5.1.1.). In addition, the health risk assessment of toxicologically relevant metabolites should result in a safe use for all relevant groups of persons. In contrast to that, a common data gap in the risk assessment arises from an unfinalized evaluation of microbial secondary metabolites. The assessment of the toxicological relevance is an important step as many microorganisms are known for their ability to produce toxic metabolites. The discussion about some of the compounds listed in this publication has delayed the approval of the respective AS for years, e.g., Beauvericin and 2,3-deepoxy-2,3-didehydro-rhizoxin (DDR). In this review we illustrate the difficulties in the assessment process and summarise results of the assessment of a number of these metabolites.

### Microbial biopesticides

The AS of microbial biopesticides can be divided into three groups i) viruses, including bacteriophages, ii) bacteria and iii) fungi [68, 110]. Their mode of action can be either bactericidal, fungicidal, insecticidal, or nematicidal. Their often high specificity to target species, reduced adverse effects compared to chemical alternatives and high biodegradability make them ideal candidates for integrated pest management (IPM), leading to a low impact on non-target organisms and the environment [8]. Currently, 9 viruses, 21 bacteria and 41 fungi are approved as PPP AS in the EU (10.11.2023).

i) Virus strains currently used as biopesticides mainly have bactericidal, anti-viral or insecticidal effects. As they do not have an own metabolism, an evaluation of metabolite production is not necessary.
ii) The bacterial species currently used as biopesticides act in a fungicidal or insecticidal way. Most of them belong to the genus *Bacillus*. If dried the *Bacillus* endospores are easily storable as an active ingredient [74]. Another form of dissemination and survival are arthrospores from *Streptomyces spp*. [113]. Nevertheless, also non-spore forming bacteria (*Pseudomonas spp*.) are currently applied as AS. Bacteria are known to produce a number of secondary metabolites which support their mode action.
iii) Filamentous fungi are usually used as insecticidal or fungicidal agents in PPP. The mass produced conidiospores are a part of their growth cycle [90]. Their cover with hydrophobins allows a good dispersal but also make recognition by the immune system difficult [7]. They contain pigments which are formed as a protective mechanism against UV radiation and give the fungal colonies their coloration [2]. Some yeast-like fungi such as *A. pullulans* or *S. cerevisiae* are also used as biopesticidal AS. Fungi can produce an enormous diversity of secondary metabolites. They use ribosomal synthesis or cluster-like non-ribosomal synthesis for production complex organic compounds [11]. The fungicidal effects base on the one hand on displacement and occupation of niches, on the other hand on the action of secondary metabolites such as antifungal peptides [103]. The reasons for non-approval of microbial biopesticides were manifold. The RMS and EFSA identified either serious data gaps in the course of the risk assessment which the EU Commission considered necessary to clarify for approval, or no sufficiently safe application was shown in the course of the assessment.

### EU guidance secondary metabolites assessment

In order to standardize the assessment of microbial SM across the EU, a guidance document was developed by the Working Group on Biopesticides, part of the European Commission's DG SANTE [93]. The guidance provides for a stepwise approach: i) Determining the assessment type, ii) Data collection, iii) Determination of MoC and finally iv) Risk Assessment. The first step is to determine whether an evaluation of the dossier according to Part A (chemical AS) or Part B (microbial AS) of Regulation No 283/2013 has to be carried out. Moreover, no assessment of metabolites is necessary in case the AS is a virus, as viruses are not expected to produce metabolites. In the second and third step, a list of all metabolites of potential (toxicological) concern is prepared. For all substances considered relevant, an exposure and toxicity assessment based on experimental, modelling and/or literature data is performed to determine whether a safe use, if necessary, by application of risk mitigation measures, can be defined. Since in most cases no reliable toxicological reference values are available and their derivation would be expensive, time consuming and including animal testing, approaches such as the concept of Threshold of Toxicological Concern (TTC) or Read-across can be used to assess the risk resulting from exposure [36, 48]. One main problem is the exposure from other natural sources, as secondary metabolites are not exclusively produced by only one certain microbial strain.

### Examples for secondary metabolites of concern

**Fig. 1:**
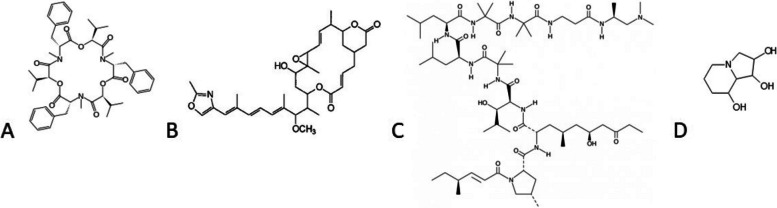
**A** Beauvericin, **B** 2,3-deepoxy-2,3-didehydro-rhizoxin (DDR) [67], **C** Leucinostatin A [9], **D** Swainsonine [97]

The data gaps and uncertainties in the assessment of microbial SM led to years-long approval procedures with much delay and additional scientific exchanges between authorities and applicants. In the following, four examples for such approval processes will be presented (Fig. 1).

#### Beauvericin

The toxicologically relevant microbial secondary metabolite Beauvericin was first described in 1969 by Hamill et al*.* as a substance toxic to *Artemia salina*, formed by *Beauveria bassiana* [57]. It is a cyclic hexadepsipeptide build by non-ribosomal peptide synthesis by various moulds, mainly *Fusarium spp*. [70]. Contaminated crops are mainly cereals and food products made from these [119]. Beauvericin is also considered a relevant contaminant in studies developing methods to prevent *Fusarium* mycotoxin contamination of food crops [29].

Currently seven strains of the entomopathogenic fungal species *Beauveria bassiana* are registered as insecticides in the EU. For detailed information on Beauvericin, for example, the work of Mallebrera et al*.* is useful [70]. Studies show equivocal genotoxic effects of Beauvericin in addition to antimicrobial, insecticidal, nematicidal, anti-inflammatory, cytotoxic, and anti-cancer effects. Moreover, natural exposure may arise from a wide range of food products. Therefore, a risk assessment was carried out on behalf of EFSA in 2014 and supplemented in 2018 with data on in vitro and in vivo genotoxicity following acute or repeated exposure [15, 31, 63, 64, 72]. The maximum acute dose used in the mouse studies was 200 mg/kg bw of Beauvericin dissolved in canola oil with 5% DMSO. The maximum dose used and administered multiple times in the subchronic study was 10 mg/kg bw per day dissolved in olive oil with 1.5% DMSO, over 42 administrations five days per week. Thus, in both experimental approaches, the dose exceeded the highest estimated human exposure for Beauvericin (0.05 μg/kg bw per day (mean) and 0.10 μg/kg bw per day (95th percentile)) [31]. None of the mice in the highest dose group of the acute study died after administration of 200 mg/kg bw Beauvericin. A large number of the effects were not concentration-dependent, possibly because of the relatively small dose groups, and must therefore be considered with caution. However, the abnormalities were observed in direct comparison with the control group. Only effects with a clear dose–response relationship were used to determine NOAEL values. Relevant findings in the acute study include micronuclei in terminally differentiated liver cells (HepaRG cells) (200 mg/kg bw dose group). The subchronic test [78, 81] showed the following results: pathology: i) ♀: cortical vacuolization of the adrenal glands, ii) ♂: Histomorphometric changes in the spleen, iii) ♂: Relative weights of heart and liver significantly decreased in the highest dose group (10 mg/kg bw per day), iv) ♂: Increased serum creatinine levels without histopathological changes in kidneys (1 mg/kg bw per day), v) altered kidney weights at all dose levels without a clear dose–response curve, vi) target tissue of Beauvericin =  > thyroid gland (histopathological changes) with higher sensitivity of male animals. Reproductive toxicity: i) Presence of endometrial cysts significantly increased, decreased myometrial area, significantly decreased serum T levels (1 mg/kg bw per day) ii) Endometrial hyperplasia (10 mg/kg bw per day), iii) Increased atrophic tubules with germ cell disorganization, iv) Tissue changes in the testes (10 mg/kg bw per day), v) Absolute and relative weight of thymus significantly increased in dams without histopathological changes (1 mg/kg bw per day, 10 mg/kg bw per day), iv) absolute and relative ovarian weights significantly increased without histopathological changes (10 mg/kg bw per day), vii) Increased severity of extra-medullary haematopoiesis in the spleen in all dams, viii) Thyroid gland with increased follicle degeneration, decreased follicle number and follicle density (10 mg/kg bw per day), thyroid stimulating hormone significantly increased (0.1 mg/kg bw per day). Genotoxicity: i) ♂: Statistically significant increased percent tail intensities in the comet assay for kidneys and duodenum (1 mg/kg bw per day). In summary, the findings indicate only a low genotoxic potential of Beauvericin and, in particular no dose–response relationship could be established [76]. The authors of the study conclude that further experiments should clarify the genotoxic potential, but also establish a NOAEL for the different dose groups based on the histopathological findings (Table 1). The data situation on the microbial SM Beauvericin demonstrates very well the risk assessment dilemma. Some studies show toxicological effects but remain equivocal. Moreover, the description as a genotoxic substance with simultaneous referencing of studies describing an anti-cancer effect, as well as publications showing synergistic effects with other pesticides [3] is contradictory. Beauvericin was assigned to substance class Cramer III (suspected significant toxicity with high relevance) in EFSA's Scientific Opinion using software evaluation (Toxtree), leading to a threshold for potentially DNA-reactive mutagens and/or carcinogens [25, 65]. Application of the TTC concept results in a threshold for oral intake of 0.0025 µg/kg bw per day, corresponding to 0.15 µg/day for a 60 kg person [31, 35, 87]. If this intake level is not exceeded, no increase in the tumour rate is to be expected according to the model. Several parameters have to be taken into account, which create additional uncertainties, when transferring the TTC threshold value for oral intake to dermal and inhalation exposure. There is no relevant first-pass detoxification effect of the liver, if any, and a possibly different sensitivity compared with the systemic effects following oral ingestion. Therefore, an adjustment of the safety factor from the results of animal studies must be made, as well as a review of the extent to which ADME from the oral studies can be extrapolated to the lung and skin organs. Studies for genotoxic substances suggest an increased sensitivity of the lung [37]. Previous publications on this topic were summarized by [35]. Arguments against the TTC concept are the resulting very low threshold value, the studies that have shown no genotoxic effect of Beauvericin, and the lack of consideration of other sources of natural contamination, or potentially occurring other genotoxic Cramer III substances, which make it possible to exhaust the threshold value even at lower concentrations of Beauvericin. The use of the TTC approach was rejected in the EFSA Conclusion on the AS *B. bassiana* strain 203. It was reasoned that the TTC approach has not previously been endorsed by risk managers for metabolite residues and is therefore not currently applicable for microbial SM [32, 44]. Finally, it was agreed that the content of 80 µg/kg Beauvericin in the product (result of the measurement of the Beauvericin content of the batch analysis) must not be exceeded [20].

**Table 1:** Profile Beauvericin

Active substance dossier	*Beauveria bassiana* (*Beauveria bassiana* strain 203, *Beauveria bassiana GHA)*
IUPAC	(3S,6R,9S,12R,15S,18R)-3,9,15-Tribenzyl-6,12,18-triisopropyl-4,10,16-trimethyl-1,7,13-trioxa-4,10,16-triazacyclooctadecane-2,5,8,11,14,17-hexone
SMILES	O = C1N(C)[C@H](C(= O)O[C@@H](C(= O)N([C@H](C(= O) O[C@@H](C(= O)N([C@H](C(= O)O[C@@H]1C(C)C)Cc2ccc cc2)C)C(C)C)Cc3ccccc3)C)C(C)C)Cc4ccccc4
Other natural sources	*Fusarium* spp. [70], filamentous fungi
Effect	Antimicrobial, insecticidal, nematicidal, anti-inflammatory, cytotoxic, anti-cancer [116, 119]
Residues in relevant crops	Cereals and cereal products
Acute toxicity	Mice oral: > 100 mg/kg bw [82] > 200 mg/kg bw [72]
Subchronic toxicity	Mice NOAEL: ♂ = 0.1 mg/kg bw/day; ♀ = 1 mg/kg bw/day Mice LOAEL: ♂ = 1 mg/kg bw/day; ♀ = 10 mg/kg bw/day [72]
Reproductive and developmental toxicity	NOAEL: Maternal = 0.1 mg/kg bw/day NOAEL: Offspring = 10 mg/kg bw/day LOAEL: Maternal = 1 mg/kg bw/day [72]
Carcinogenicity and genotoxicity	low genotoxic potential [72]

#### 2,3-deepoxy-2,3-didehydro-rhizoxin (DDR)

The microbial secondary metabolite 2,3-deepoxy-2,3-didehydro-rhizoxin (DDR) (Table 2) was identified as a potential genotoxic substance in the AS dossier for the bacterial fungicide *Pseudomonas chlororaphis* strain MA 342. The metabolite is synthesized stepwise via non-ribosomal protein synthesis in the cell. The underlying trans-AT NRPS-PKS gene cluster was identified for the metabolite rhizoxin in the fungal endosymbiont *Burkholderia rhizoxina* [86]. The possible toxicological effects of DDR are unclear according to the EFSA statement. There, the experts of the "Standing working group on genotoxicity of the Scientific Committee" doubt the validity of the results of the in vitro study [83] to determine the aneugenic effect of DDR, due to the inadequate reporting of the results. The subsequent in vivo follow-up study [33] then no longer allowed a statement on the inhibition of mitosis [33] and the crucial effect as an inhibitor of microtubules could not be reliably shown. Thus, it is clear that DDR induces chromosomal damage, but whether it is induced by an aneugenic or a clastogenic mechanism is important for deriving a toxicological reference value or not. Therefore, only the application of the TTC concept remains for the derivation of a toxicological reference value for the assessment of the exposure to a potentially genotoxic metabolite (0.0025 µg/kg bw/day). The limit of quantification (LoQ) was taken as the maximum content of DDR to be assumed in the TGAI. Thus, together with the applied amount of AS in the product, the exposure for operators and workers can be calculated and, depending on the application method, also for bystanders. In this way, the TTC concept is again applied beyond the assessment of oral exposure. A guidance document for the calculation was published in 2022 [46]. Using both EUROPOEM and the German Model as model scenarios, the exposure to DDR for bystanders and residents was below the TTC reference value for the representative uses of the application. If the TTC value is used as the AOEL for the operator, it is only possible to fall below this value assuming that protective equipment and gloves are worn, depending on the model. But similar to Beauvericin, the use of the TTC concept is questionable.

**Table 2:** Profile DDR

Active substance dossier	*Pseudomonas chlororaphis* strain MA 342
IUPAC	2,3-deepoxy-2,3-didehydro-rhizoxin
SMILES	COC(/C(C) = C/C = C/C(C) = C/c1coc(C)n1)C(C)C3CC(O)C2(C)OC2/ C = C/C(C)C4CC(C/C = C/C(= O)O3)CC(= O)O4
Other natural sources	*Pseudomonas* spp.,* Burkholderia rhizoxinica*
Effect	Insecticide, nematicide
Residues in relevant crops	unknown
Acute toxicity	unknown
Short term toxicity	unknown
Reproductive and developmental toxicity	unknown
Carcinogenicity and genotoxicity	Chromosome damage, unclear effect [33]

Consumer exposure can also be calculated based on a theoretical worst-case amount of applied metabolite and it is related to the lowest expected crop harvest. In addition, using the EFSA PRIMo—Pesticide Residue Intake Model [45, 46], the average amount of the respective crop consumed is taken into account as the basis to match acute and chronic exposure to PPP residues in combination with toxicological reference values. With an ADI of 0.0025 µg/kg bw/day and the assumption of a DDR content in wheat of 1 µg/kg (LoQ), the TTC reference value was exceeded multiple times. The inclusion of a degradation rate to the calculation might give a more realistic picture of the actual residues but is only applicable if the in situ production of DDR is neglected. The RMS and Co-RMS disagreed with the presentation in the EFSA publication arguing that DDR has a maximum half-life of 91.2 h (pH 7, 10 °C). The concentrations of DDR would decrease so much, depending on a storage time of the PPP at least one week before application, that the assumption of contamination at the LoQ level of 1 µg DDR per kg wheat is unrealistic.

To comprehensively assess the risk of exposure a possible translocation of the bacterium within the plant and thus potentially also the translocation of the metabolite DDR has to be considered [33]. Seed treatment of cereals and peas leads to a distribution of the microorganisms within the plant of up to 10^5^ CFU/g plant material, potentially producing DDR [33].

EFSA had made clear in 2020 that a translocation of *P. chlororaphis* MA342 in the plant is likely and therefore a formation of the metabolite DDR in the plant cannot be excluded [33]. The EC, in turn, estimated the potential exposure to the metabolite DDR from an application of *P. chlororaphis* in seed treatment as negligible, because the degradation studies showed a short half-life. Based on the time required for seed storage, crop growth and subsequent storage and transport of crops until consumption no residues would then be expected. The situation is different for the case of the spray application also applied for, which have not been approved. Here, other exposure scenarios and time frames need to be considered, resulting in no safe use. for this reason [42].

#### Leucinostatins

During the assessment of the AS *Purpureocillium lilacinum* PL11, the microbial secondary metabolite Leucinostatin was discussed as an acutely toxic metabolite of concern for human health as the metabolites Leucinostatin A and Leucinostatin B had been detected in the AS. In the acute animal studies required by EU Regulation 283/2013, there were no adverse effects identified for Leucinostatins in the different exposure routes [43]. However, several studies had demonstrated high toxicity after oral and intraperitoneal application of Leucinostatin A and B in mice (Table 3) resulting in a classification as orally toxic, Cat. 2 with the H-phrase H300 according to CLP guidelines [38] However, in the absence of toxicological reference values for acute and chronic exposure the risk assessment for operator, worker, bystander, resident and consumer could not be finalised [38]. For the operator risk assessment, wearing protective equipment to reduce dermal and inhalation exposure was considered sufficient to protect against Leucinostatins in the product. However, as in situ formation of Leucinostatins is unclear and no degradation rates have been reported, open questions in the field of groundwater exposure, persistence in soil and residues in crops remained open. In an EFSA expert meeting, it was agreed that a level of 0.1% Leucinostatin in the TGAI should not be exceeded [43]. Available analyses of the TGAI showed that this value is complied with. The EC however did not follow the proposed restriction in the review report. Noteworthy, the product application in this case was for drip irrigation use only. Therefore, dermal and inhalation exposure is lower than for a spray application that might be applied for in the zonal authorisation process in the future.

**Table 3:** Profile Leucinostatin

Active substance dossier	*Purpureocillium lilacinum* PL 11
IUPAC	N-[1-[[1-[[1-[[1-[[1-[[1-[[1-[[3-[1-(dimethylamino)propan-2-ylamino]-3-oxopropyl]amino]-2-methyl-1-oxopropan-2-yl]amino]-2-methyl-1-oxopropan-2-yl]amino]-4-methyl-1-oxopentan-2-yl]amino]-4-methyl-1-oxopentan-2-yl]amino]-2-methyl-1-oxopropan-2-yl]amino]-3-hydroxy-4-methyl-1-oxopentan-2-yl]amino]-6-hydroxy-4-methyl-1,8-dioxodecan-2-yl]-4-methyl-1-[(E)-4-methylhex-2-enoyl]pyrrolidine-2-carboxamide
SMILES	CCC(C)C = CC(= O)N1CC(CC1C(= O)NC(CC(C)CC(CC(= O)CC)O)C (= O)NC(C(C(C)C)O)C(= O)NC(C)(C)C(= O)NC(CC(C)C)C(= O)NC(C C(C)C)C(= O)NC(C)(C)C(= O)NC(C)(C)C(= O)NCCC(= O)NC(C)CN(C) C)C
Other natural sources	*Paecilomyces, Purpureocillium lilacinum*
Effect	Antibiotic [5], Pore former [50]
Residues in relevant crops s	unknown
Acute toxicity	Mice oral: Leucinostatin A:5.4 mg/kg bw [51] Leucinostatin B:6.3 mg/kg bw [51] Mice intraperitoneal: Leucinostatin: 1.6 mg/kg bw [5] Leucinostatin A und B: 1,8 mg/kg bw [51] Leucinostatin A-HCl: 1.2 mg/kg bw [95]
Subchronic toxicity	unknown
Reproductive and developmental toxicity	unknown
Carcinogenicity and genotoxicity	unknown

#### Swainsonine

A variety of plants from the genera *Tragacanth* (*Astragalus*), *Swainsona*, and *Oxytropis* synthesize the secondary metabolite Swainsonine (Table 4), collectively known as locoweeds. The term is derived from the Spanish adjective "*loco*" which means "mad, crazy, insane". When grazing animals consume feed contaminated with Swainsonine over several weeks, neurological disorders may develop, which are summarized under the term locoism [117]. The sensitivity of different animal species varies. Horses are the most sensitive, followed in descending order by sheep, cattle, deer, and rodents [23]. In experiments with mouse models, 4 mg/kg bw of Swainsonine was administered intraperitoneal for seven consecutive days or 10 μg/ml was administered in drinking water for four weeks [104, 115]. In horses, symptoms appear at the earliest after two or more weeks after ingestion, in ruminants after at least four weeks. Furthermore, synthesis by filamentous fungi of the genera *Metarhizium*, *Rhizoctonia*, *Alternaria sect*., *Undifilum*, and *Embellisia* also occurs [94]. When representatives of these genera grow on or as endophytes in plants, they may be responsible for contamination of the plant with Swainsonine [26, 56, 105]. Swainsonine is a water-soluble indolizidine alkaloid that is rapidly absorbed in the animal digestive tract and excreted in urine, manure, and milk. The formed cation blocks the Golgi alpha-mannosidase II (MAN2A1), the lysosomal alpha-mannosidase (MAN2B1), and the endoplasmic reticulum cytoplasmic alpha-mannosidase (MAN2C1) [18]. Similar, gene mutations in the alpha-mannosidase-gene lead to the rare disease alpha-mannosidosis in humans [84]. Reduced mannosidases activity leads to loss of lysosomal hydrolysis, hindering the assembly of mannose-containing oligosaccharides and the synthesis of glycoproteins and finally leading to the accumulation of mannose-containing oligosaccharide building blocks [94, 102]. This disrupts cellular processes such as cell–cell communication, cell movement, cellular adhesion, and intracellular trafficking. Furthermore, vacuolization, especially in neurons, and cellular vascular degeneration of most tissues result from this lysosomal storage disorder. Effects are visible in the reproductive, nervous, endocrine, and immune system [17]. Repeated ingestion of low doses primarily results in decreased weight gain in grazing animals. At higher doses, symptoms at the onset of intoxication include depression, anorexia, and weight loss [23]. If dietary ingestion of Swainsonine persists, other neurological symptoms including increased listless behaviour, staggering gait, tremors, or ataxia occur. General effects are a dull coat, decreased libido, water belly, cardiovascular disease and ultimately death [17, 23]. Most signs of poisoning are reversible. However, behavioural abnormalities based on brain damage are irreversible. Studies in pregnant and non-pregnant grazing animals have shown that ingestion of plants containing Swainsonine leads to reproductive disorders by reducing serum progesterone concentrations. This results in ovarian dysfunction with delayed oestrus and prolonged oestrous cycle. Ultimately, delayed conception, abortions, and foetal malformations occur [85, 118]. Interestingly, in murine tumour models, Swainsonine shows reduction in tumour cell metastasis, enhancement of cellular immune response, and reduction in solid tumour growth [104]. Ren et al*.* provide a good summary on anti-tumour effects [94]. Results in animal studies led to phase I and phase 1B clinical trials in patients with solid tumours and hematologic [53, 104]. To determine the quantitative and qualitative toxicity of Swainsonine in patients, 19 patients in the phase I study underwent continuous *i.v.* infusion for 5 days, repeated in 31 cycles at 28-day intervals. The dose was increased in increments of 100 μg/kg bw/day from 50 to 550 μg/kg bw/day. The maximum tolerated doses were 550 and 450 μg/kg bw/day, respectively, under this dose regimen. Common side effects included edema, mild hepatic dysfunction, an increase in serum amylase, and a decrease in serum retinol. The clearance and serum half-life of Swainsonine were determined to be approximately 2 ml/h*kg bw and 0.5 days, respectively, and toxicity was determined to be low [53]. The subsequent Phase IB study was designed to investigate the pharmacokinetics and toxicity of bi-weekly oral Swainsonine at increasing doses (50—600 μg/kg bw). Based on serum aspartate aminotransferase abnormalities and dyspnoea, the maximum tolerated dose was set at 300 μg/kg bw/day. Other side effects to those observed in the intravenous study included fatigue, anorexia, dyspnoea, and abdominal pain. The study authors concluded that a dose of 150 μg/kg bw/day is tolerated with chronic intermittent oral administration, but further studies should investigate the efficacy of lower doses [54]. Phase I clinical trials have been conducted with severely ill individuals, and it is questionable what can be inferred for healthy individuals and chronic exposure. There are few studies describing targeted chronic exposure of grazing animals to Swainsonine. Feeding 1 mg/kg bw to sheep for 30 days resulted in accumulation of Swainsonine in the body and demonstrated a half-life in skeletal muscle, heart, brain, and serum of less than 20 h. The accumulation in liver, spleen, kidney and pancreas was about tenfold greater, which could be explained by a higher half-life of about 60 h in these tissues [108]. Other studies showed that short-term (< 28 days) consumption of 0.2 mg/kg Swainsonine or less may be effective as a minimum in animals [107, 109]. Overall, it appears that daily doses in the two-digit microgram range can be assumed for chronic effects in adults due to rapid excretion. Due to the influence on developmental processes, the ADI for children must certainly be set lower. The attempt to derive an ADI for adults based on the Stegelmeier et al*.* study resulted in a NOAEL of 0.05 mg/kg bw/day. The calculation leads to an ADI of 0.17 µg/kg bw/day, taking into account an interspecies factor of 10, an intraspecies factor of 10, and a factor of 3 to derive from subchronic to chronic exposure [107].

**Table 4:** Profile Swainsonine

Active substance dossier	*Metarhizium brunneum* strain Ma 43
IUPAC	(1S,2R,8R,8aR)-octahydroindolizine-1,2,8-triol
SMILES	O[C@H]1[C@@]2(N(C[C@H]1O)CCC[C@H]2O)[H]
Other natural sources	*Rhizoctonia leguminicola*, plants: *Swainsona canescens*, *Astragalus lentiginosus, Oxytropis sericea*
Effect	unknown
Residues in relevant crops	weed
Acute toxicity	unknown
Subchronic toxicity	Grazing animals (< 28 days) consumption of ≤ 0.2 mg Swainsonine effective [107, 109]
Reproductive and developmental toxicity	Disruption of ovarian function with delayed oestrus and prolonged oestrous cycle. Ultimately, delayed conception, abortions, and foetal malformations occur [83, 116]
Carcinogenicity and genotoxicity	unknown

$$ADI=\frac{50 \mu g {*kg}^{-1}* {day}^{-1}}{10*10*3} =0.17 \mu g*{kg}^{-1} {*day}^{-1}$$

For comparison with the ADI, the dossier of *M. anisopliae* lacks information on the concentration of Swainsonine in the product, or the analysis of residues in treated crops. At least in the product, an analysis with a limit of detection (LoD) of 30 ppm or 30 mg/kg was performed and no Swainsonine was detectable above this concentration. Taking into account the intended application rates, the expected amount of field crop per hectare and a maximum content of 30 mg/kg Swainsonine in the product, a worst-case calculation for the expected Swainsonine residues at harvest can be performed. These values can in turn be used as input values for consumption models to calculate the compliance with the ADI. However, other natural sources as well as in situ production of the metabolite after application are not considered here.

Besides the metabolites mentioned here in detail, other microbial secondary metabolites are relevant in the PPP and TGAI assessment like e.g. 6-pentyl-2H-pyran-2-one, kojic acid, surfactins, iturins, fengycins and trichothecenes [13, 61, 66, 71, 88, 89]. Most secondary metabolites have in common that their data set was limited and did not allow to finalise the risk assessment. Therefore, risk assessors are in need of new strategies to handle this data point. Even if testing AS extracts in a tiered approach, the effects explored need to be narrowed down to a single substance, making more tests necessary best without the use of animals and following the 3R-principle [69]. The more complex the effects of a secondary metabolite are, e.g. neurotoxic or endocrine disturbance, the more complex is the test methodology.

### Testing and evaluation strategy for risk assessment of microbial secondary metabolites

In the section above it has become evident, that often a lot of information is missing to conclude on potential health risks of microbial secondary metabolites in PPP. New Approach Methodologies (NAMs) refer to new methods and approaches for assessing the hazards that may result from human and animal exposure to chemical substances [30]. NAMs contribute to Next Generation Risk Assessment (NGRA), which includes integrated approaches to testing and assessment (IATA) as a framework [100, 111]. Practically, scientific methods can help to combine existing and new information using a variety of testing strategies. In this regard, the assessment of microbial secondary metabolites can benefit from the general advancement in the field of chemical risk assessment to address issues of consistency and transferability of results, sustainability and cost of research, and ethical reasons [52]. By now, alternative *in vitro*, *in silico*, or *in chemico* methods are not yet fully established and validated for some endpoints, such as carcinogenicity or reproductive toxicity [59]. One of the major hurdles is the transferability of results from, for example, *in vitro* cell assays to a whole organism [98]. To overcome these problems, stepwise approaches in adverse outcome pathways (AOP) are used to build a risk assessment from a variety of independent results [4, 47]. The OECD, among others, is enabling globally harmonised principles and supports the development of the NGRA, also for microbial pesticides [16, 77]. Additionally, the EU through EFSA, the MS and the COM is also involved and develops a framework for the application of AOPs in NAMs [101, 112].

Even if for more complex endpoints NGRA is not yet feasible, there are numerous projects working in this direction (e.g. PARC) [73]. There are examples for endpoints, where in vitro methods are already fully implemented in RA, such as for genotoxicity or skin sensitization. For skin sensitization testing by using an IATA and in vitro methods an OECD Test guideline is already available [75, 79, 80].

Such a tiered model may also be established for the further development of NAMs in the risk assessment of microbial SM and further microorganism. It can be structured similar to the assessment stages in the microbial secondary metabolite guidance document [93]. The first stage would be the identification of microbial secondary metabolites potentially produced by the microorganism using *omics* [47]. After sequencing the genome, it can be screened for genes for known secondary metabolites in databases such as AntiSmash, Bactibase, and ClusterMine360 (genomics) [10, 19]. A good overview of available databases can be found at https://www.secondarymetabolites.org/databases/. The increasing knowledge about the genetic background for the synthesis of numerous secondary metabolites is mainly due to the interest of the pharmaceutical industry in new drugs [58, 91]. Evidence that genes relevant to synthesis are not present in the genome of the strain is unequivocal, and no further consideration on a potential hazard is required. If the respective genes are detected, an analysis of the TGAI can then show whether these genes were active under manufacturing conditions of the AS and if the metabolite is present in the AS or the product. Another method for identifying metabolites produced under specific conditions is metabolomics [12]. This method can be applied to examine which metabolites are produced during the production of microbial TGAI [24]. A risk assessment based on the hazard (toxicological properties of the substance) and the expected exposure can be made according to the concentration of the substance in the TGAI. It has to be kept in mind that also in situ production of the metabolite might be relevant even though only a residue trial would elucidate relevant residues on crops. Other applications for the described methods are possible e.g. for in situ production, as well as the question of which influence the application of the microorganism has on the soil microbiome [49]. One can also use metabolomics to determine the microbial biodiversity of, for example, the soil [106].

The second stage of a tiered model would be the identification of secondary metabolites with potential concern for human health. Here, in silico analysis in QSAR models combined with testing individual available substances in AOPs for their toxicological relevance is suggested [28, 96]. Theoretically, different endpoints such as acute toxicity, genotoxicity, or chronic toxicity can be addressed [55, 58, 99]. By using extracts of the TGAI, mixtures of formed metabolites can be analysed. A good overview of existing AOPs and those under development is provided by the AOPwiki website (https://aopwiki.org/). Another approach for the second stage would be to investigate which of the predicted metabolites are present in relevant amounts in the TGAI. Thus, for example, using the TTC concept, substances can be excluded from further toxicological analysis based on their low concentration in the TGAI [34, 35].

The subsequent third step would be the determination of threshold values for substances determined as being of concern, especially if the derivation by means of TTC is not convincing. However, this would only be the case if the secondary metabolite is present in relevant amounts in the TGAI, is of toxicological concern and therefore a threshold value is needed for the quantitative risk assessment.

The main difference to the current approach by using the NGRA is the consideration of (almost) all produced microbial secondary metabolites and the attempt to perform a risk assessment even without existing toxicological prior knowledge from literature or studies about the SM under assessment.

## Conclusion and outlook

Scientific risk assessment of microbial PPPs is caught in a "nice to know—need to know" dilemma, especially in assessing the toxicological relevance of SM. Are the data rather scientifically interesting (nice to know) or really necessary for the risk assessment process (need to know)? It is sometimes difficult in data assessment to weigh the depth to which it is required to answer a question in order to have sufficient knowledge for a regulatory assessment of the hazard and the derived risk. An example is the handling of data gaps on the production of SM by a microorganism species that is applied for approval as a PPP AS. The application of the guideline results in a clear definition of which microbial secondary metabolites have to be evaluated. A major factor of uncertainty remains the lack of knowledge regarding the toxicological relevance of the SM for human health, threshold values for many microbial metabolites and information on other natural sources of the SM. For a newly isolated strain, there is usually no information (yet) available in the freely available literature. However, there may be publications on the microbial species as part of the natural microbial flora of the human body, or examples of human and animal exposure without adverse effects. Based on such information, it may be decided on a case-by-case basis that there are probably no metabolites of potential concern that need to be considered in the risk assessment. A complex overview in form of a decision tree for the assessment of the human health relevance of a natural substance like microbial AS was proposed by industry experts of the field [14]. However, it remains questionable to what extent absence of data in the literature is comparable to an absence of hazard.

Another approach would be a technically simple analysis for the potential to form secondary metabolites by combined in NGRA genome sequencing and bioinformatics. Bioinformatic databases are available to identify common metabolites of potential concern (https://www.secondarymetabolites.org/databases/) [10]. Importantly, this includes genes for the synthetic pathways of species-specific known metabolites. In silico analyses are of limited help for complex chemical structures and a read across is also only possible if the database used cover the necessary background as for example suggested based on results from the EUToxRisk project [36].

To sum up, as most SM are involved in the mode of action, at least some additional exposure from the use of a microbial pesticide is expected compared to the natural background as otherwise the efficacy of the application is questionable. Testing the PPP for SM is possible and, if necessary, an adjustment of the production process to reduce the SM in the TGAI has to be considered as well as risk mitigation measures during and after application. However, in situ production cannot be predicted and only hardly estimated. It is under debate, however, to what extent the in situ production is significant. There are indeed very critical and stable substances such as aflatoxins but most microbial secondary metabolites are only formed by the microorganism under certain conditions as e.g. direct contact with the pest. The available studies show that, as a rule, the concentration of the microorganism decreases after its application, because it is competitive only in its biological niches. Then, only there, after certain exogenous stimuli, the formation of the secondary metabolites takes place. This includes a certain bacterial concentration as required in quorum sensing or other environmental stimuli [1, 27]. It is questionable to what extent in situ production following the release of a microorganism plays a role for the risk assessment. Assuming microbial presence in soil of up to 10^10^ bacteria per gram with species richness of 4 × 10^3^ to 5 × 10^4^ species, it is likely that an additional species / strain is of little consequence [92]. The prerequisite is, of course, that it does not act as a pathogen or pose another hazard. This is then also true for the metabolites it produces in situ [62]. Furthermore, a large number of molecules are produced by more than one microbial species, so there is a natural background exposure to metabolites [114]. One way to overcome the limitation of insufficient toxicological data for a chemical substance to be assessed is cross-reading, e.g. based on structural similarities as in the TTC concept. The classification into Cramer substance classes allows the use of a general threshold value for a group of substances. Unfortunately, the TTC concept is to be applied only for the AS itself but not for microbial metabolites, is only developed to cover the oral intake and it leads to low threshold values that can already be exceeded by the natural background. Therefore, the focus of the assessment should be on the level of secondary microbial MoC in the TGAI or PPP. However, these must be reliably identified and analysed.

Microbial SM are often key contributors to the plant protective mode of action. The aim of risk assessment is to protect operators, worker, bystanders and consumers from exposure above an acceptable level, i.e. from risks for human health. Further research and development in this area is needed, especially to establish methods such as *omics* analysis or undirected MS-analysis to establish a metabolite profile of a microorganism as a standard in assessment. Further development of NAMs, especially in silico methods for more complex molecules and a read across approach can help in assessing the risk from a microbial SM identified as relevant impurity in a TGAI or the product. This may result in a more efficient risk assessment with fewer data gaps, for the benefit of risk assessors, risk managers, operators, workers, bystanders and consumers.

## Data Availability

No datasets were generated or analysed during the current study.
